# A Practical Approach to Combined Transcatheter Mitral and Tricuspid Valve Intervention

**DOI:** 10.3389/fcvm.2021.706123

**Published:** 2021-10-13

**Authors:** Lucas Burke, Magdi Hassanin, Geraldine Ong, Neil Fam

**Affiliations:** St. Michael's Hospital, Toronto, ON, Canada

**Keywords:** mitral regugitation, tricuspid regurgitation, heart failure, transcatheter, combined approach

## Abstract

Concomitant tricuspid regurgitation (TR) is common in patients with mitral regurgitation (MR). While current guidelines recommend repair of both valves at the time of surgery when feasible, high risk patients are often undertreated, leading to significant morbidity and mortality. With advances in transcatheter edge-to-edge repair (TEER) devices and technique, combined TEER for treating significant MR and TR has emerged as a new tool for heart failure management. Recent evidence has shed light on which patients with severe TR should be targeted for transcatheter intervention either in isolation or in combination with a MV TEER procedure and allows for expanded treatment options in patients who otherwise would be limited to medical management. Technological advancements remain ahead of robust clinical data, and thus randomized clinical studies in patients with severe MR and TR will be instrumental in determining the best approach in treating these patients with transcatheter therapies.

## Introduction

Transcatheter edge-to-edge repair (TEER) with the MitraClip (Abbott Vascular, Santa Clara, USA) has been demonstrated to be safe and effective in treating severe mitral regurgitation (MR) of both degenerative and functional etiologies (1, 2). Similarly, TEER using the PASCAL system (Edwards Lifesciences, Irvine, USA) is associated with excellent survival, improved functional status and quality of life in MR patients at 1 year (3). Tricuspid valve (TV) TEER has been shown to be safe and effective using the TriClip device (Abbott Vascular, Santa Clara, USA) (4) and an early feasibility study using PASCAL for TV TEER recently reported encouraging 30-day outcomes (5).

Functional (secondary) tricuspid regurgitation (TR) is the most frequently encountered etiology for TR and refers to regurgitation not related to primary organic tricuspid valve disease (6). Multiple studies have shown increasing TR severity is associated with worse survival regardless of age, left (LV) or right ventricular (RV) dysfunction and pulmonary hypertension (7). Both late residual TR seen after left-sided valve valve surgery (8) and isolated severe TR (6) carry excess mortality and morbidity.

Significant TR may not improve predictably after treatment of the left-sided valve lesion and reduced RV afterload; thus, TR should be managed as part of the index procedure (8–12). MV repair alone is often associated with an initial improvement in TR and RV function. However, the result may be temporary, with frequent recurrence or progression of TR and most of the available data comes from surgical literature (8–12). In contrast, concomitant TV repair effectively and durably eliminates severe TR and improves RV function, supporting a more aggressive approach to important functional TR (12, 13). There is emerging data on the impact of isolated MV TEER and to a lesser extent combined MV and TV TEER on clinical outcomes in patients with both severe MR and TR (Table 1). The aim of this article is to review the existing data on feasibility and benefits of combined transcatheter mitral and tricuspid repair and highlight the important considerations for patient selection and procedural success.

**Table 1:** Survival and recurrent TR rates of combined mitral and tricuspid repair versus isolated Mitral repair.^a^

	**Type of repair**	**Baseline TR grade**						**Proportion of TR at 1 year**						**Mortality rate**			
								**No/mild**		**Moderate**	**Severe/massive**						***P*** **-value**
COAPT trial. (14) ≤ mild TR *n* = 501 ≥ moderate TR *n* = 98	TEER MITRAL	None (0) *n* (%)	Mild (+1) *n* (%)	Moderate (+2) *n* (%)	Mod-severe (+3)	Severe +4		NA		NA	NA			≤ mild TR *n* = 501 (%)	≥ moderate TR *n* = 98 (%)		0.006
		12/501 (2.4)	489/501 (97.6)	92/98 (93.9)	5/98 (5.1)	1/98 (1)								97 (19.7)	29 (31.1)		
GRASP registry (15)^b^	TEER MITRAL	No/mild *n* (%)			99 (67.8)			70 (70.7)		3 (3)	0			No/mild TR	Moderate TR	Severe/ massive TR	
		Moderate/Severe *n* (%)			47 (32.2)			18 (18.2)		8 (8.1)	0			7 (8.8)	5 (16.1)		0.213
Mehr et al. (16)	TEER MITRAL *N* = 106 (%)	None/mild		Moderate	Severe	Massive		NA		NA	NA			33 (34.0)			0.002
		0		0	106 (100)	NA											
	TEER Combined *N* = 122 (%)	0		0	69 (56.6)	53 (43.4)		49 (40.2)		48 (39.3)	25 (20.5)			20 (16.4)			
Kavsur et al. (17)^c^	TEER MITRAL *N* = 531 (%)	220 (41)		209 (39)	102 (19)			176/346 (51%)		115/346 (33)	55/346 (16)			NA	NA	NA	
TRAMI registry (18)	TEER MITRAL *n* = 766	334 (43.6)		326 (42.6)	106 (13.8)	NA		NA		NA	NA			No/mild TR	Moderate TR	Severe TR	<0.001
														14.6%	21.0%	34%	
Dreyfus et al. (10)^d^ (Surgical repair)	TR Grade =	0	1	2	3	4	*P*- value	0	1	2	3	4	*P*- value	3 ys	5 ys	10 ys	NS
	MVr *n* = 163 (52.4%)	54 (33.1)	102 (62.6)	7 (4.3)	0	0	0.02	8 (5)	33 (20.2)	67 (41.1)	40 (24.5)	15 (9.2)	<0.001	2.7%	3.8%	14.5%	
		Mean TR grade = 0.7 ± 0.5						Mean TR grade =2.1 ± 1.0									
	Combined MVr + TVr 148 (47.6%)	38 (25.7)	92 (62.1)	16 (10.8)	2 (1.4)	0		102 (68.9)	41 (27.8)	4 (2.7)	1 (0.6)	0		1.5%	1.5%	9.7%	
		Mean TR grade = 0.9 ± 0.6						Mean TR grade = 0.4 ± 0.6									

a *1-year for most studies. In some studies, adjusted to severity of TR*.

b *Total of 99 patients analyzed for grade of TR at 1 year*.

c *165 patients with one missing follow-up echocardiography were excluded from 1 year follow up of TR proportion*.

d *Follow up data of TR over period of 2-8 years. Survival/mortality rate presented calculated at 3, 5, -and 10-years post-operative*.

## Physiopathology of Combined Mitral and Tricuspid Regurgitation

MR is either functional, secondary to annular dilatation and leaflet tethering as seen with ischemic or dilated cardiomyopathies and atrial fibrillation, or primary in the setting of mitral valve apparatus dysfunction in rheumatic heart disease, myxomatous degeneration, infective endocarditis (IE), among other etiologies. Most cases of significant TR are secondary to dilatation of the tricuspid annulus and/or leaflet tethering due to RV remodeling in the setting of pressure or volume overload such as pulmonary hypertension, dilated cardiomyopathies, or atrial fibrillation (19–21). Primary etiologies of TR include rheumatic, IE, congenital (Ebstein's), myxomatous, blunt chest trauma, carcinoid, drugs, and radiation. A growing number of patients develop significant TR from iatrogenic etiologies such as intracardiac device leads and endomyocardial biopsies (22–24).

## Imaging Evaluation of Combined Valve Disease

### Identifying Etiology and Preprocedural Planning

Echocardiography remains the critical imaging modality in selecting patients for combined MV and TV TEER, which includes complete transthoracic echocardiography (TTE) and transesophageal echocardiographic (TEE) views according to recommendations (25) for a comprehensive cardiac assessment (26). Accurate evaluation of MR and TR severity using quantitative parameters is recommended (27). Quantitative assessment of TR severity has less established cut-off values in comparison with MR severity assessment (27, 28). Hahn et al. has suggested a new 5-grade scale for TR severity grading, expanded to include massive and torrential TR (28). In comparison to imaging the MV, TV imaging is more challenging as the tricuspid leaflets are thinner and present with a variety of morphological variants. Additionally, the TV is an anterior structure in the field far from the TEE probe. While TEE is used for defining the precise mechanism of TR, patient selection and procedural guidance, TTE is essential for assessment of the TV and quantifying RV function under basal conditions (29).

**Figure 1:**
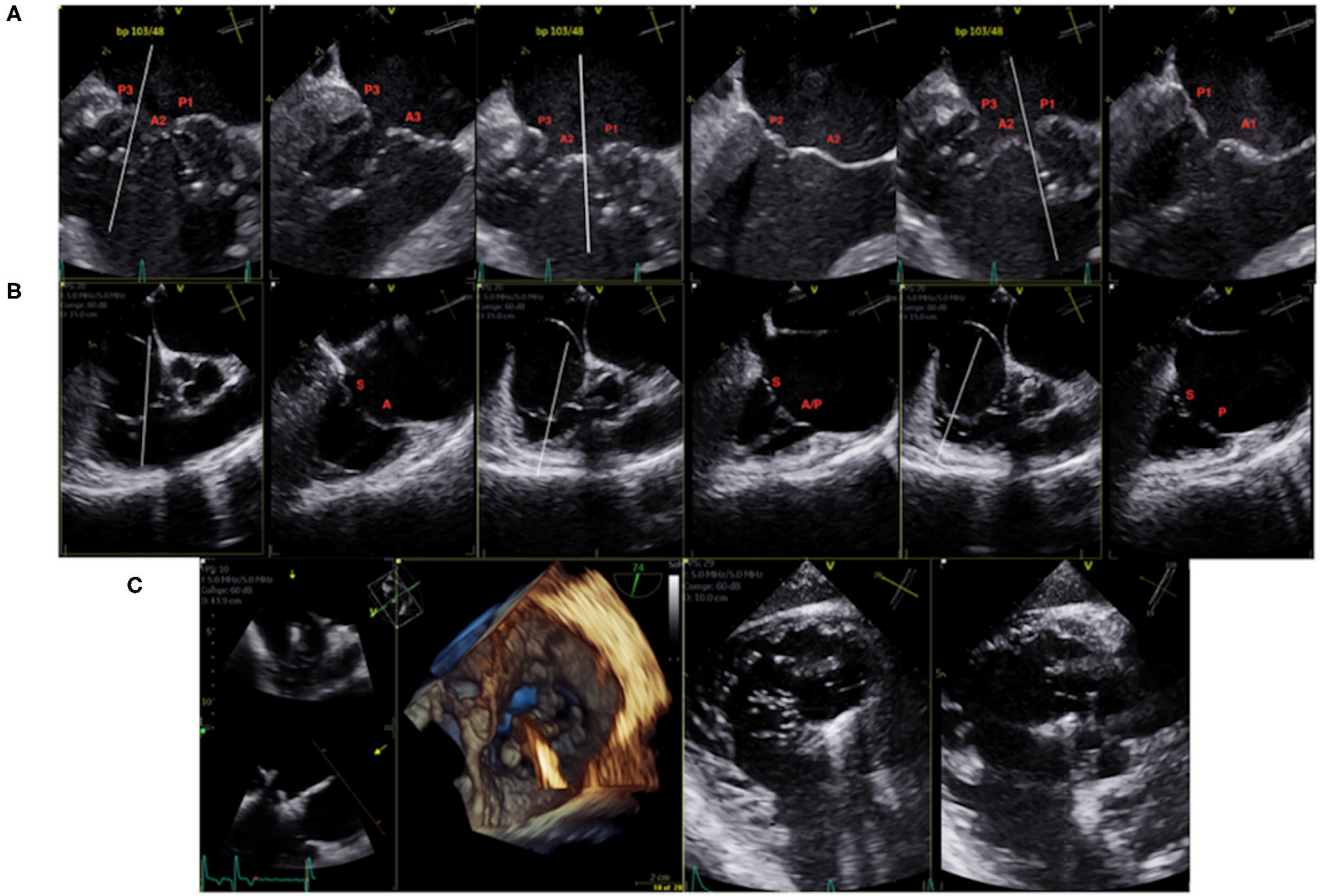
**(A)** 2D TEE X-plane of the mitral valve pre-procedural mapping (bi-commissural view). **(B)** 2D TEE X-plane of the tricuspid valve pre-procedural mapping (bi-commissural view). **(C)** TEE 3D and trans-gastric intraprocedural guidance of clip insertion in the anterior/septal position.

The use of advanced echocardiography features such as multiplanar views and 3-dimensional imaging has significantly improved the accuracy of the diagnosis and invasive management of valvular disease. Using the X-plane mode, two simultaneous orthogonal planes are obtained, allowing visualization of morphological details and precise determination of cardiac valvular lesions (Figures 1A,B). While echocardiographic assessment is sufficient for planning of TEER procedures, cardiac CT is required when planning transcatheter valve replacement procedures of the MV (TMVR) or TV (TTVR). CT imaging is crucial for TMVR as it better assesses the dimensions and geometry of the mitral annulus, determines optimal device landing zones and helps to predict the risk of left ventricular outflow tract obstruction and paravalvular leak. Equally, utilizing CT for planning TTVR procedures allows for accurate assessment of the tricuspid annulus, RA and RV dimensions and inferior vena cava-TV relationship (30).

## Intraprocedural Guidance

Interventional echocardiography is an emerging and growing field and is essential for the guidance of many transcatheter structural heart procedures. Similar to other transcatheter valve interventions, combined MV and TV TEER procedural success relies on adequate visualization of the cardiac structures and skilled intraprocedural guidance, with a focus on device positioning and successful grasping of the MV and TV leaflets. Imaging quality will depend not only on numerous patient characteristics (e.g., shadowing from prosthetic valves, hypertrophied interatrial septum, massive atria, horizontal orientation of the heart, chest/spine deformities, esophageal anatomy/pathologies), but also on the device used for repair.

For streamlined intraprocedural guidance, the rapid transition between mid-esophageal multiplanar views to transgastric to 3-D imaging of the valve is required. Furthermore, standardizing imaging views of the valves allows for efficient communication between the interventionalist and the interventional echocardiographer, increasing the rate of procedural success while decreasing procedure time. In general, transcatheter TV procedures are more challenging than those on the MV as the TV leaflets are often difficult to simultaneously visualize on 2D echocardiography (31), thus 3D echocardiography and transgastric views are mandatory for effective procedural guidance (Figure 1C). Lower-esophageal views may also be helpful. Many procedural imaging steps for MV TEER also apply to TV TEER, however transgastric views in particular are essential to guide TV TEER (32) as they provide excellent visualization of TV leaflet morphology, coaptation gaps, device landing zones, and location of the predominant TR jet (32, 33). Furthermore, transgastric views provide confirmation of complete leaflet insertion into the center of the clip prior to device deployment. When TEE is inconclusive, on-table TTE imaging may provide confirmation of adequate leaflet insertion. More recently, intracardiac echocardiography has been used for isolated TEER procedures on both the mitral (34) and tricuspid (35, 36) valves. The use of 4D ICE for mitral procedures is useful in patients with contraindications to TEE or in whom the risk of general anesthesia is too high, or specifically in the case of TV interventions, when TEE imaging is suboptimal.

## Postprocedural Assessment

Postprocedural assessment by TEE in the immediate post-deployment phase of the procedure is crucial in assessing the reduction in regurgitation severity and to rule out procedural complications (e.g., single leaflet device attachment (SLDA) or pericardial effusion). Follow up postprocedural TTE targets the assessment of long term success in terms of sustained reduction of regurgitation, ventricular reverse remodeling, reduction in afterload and stability of the device (37).

## Evidence Supporting Combined Surgical Intervention

In the presence of concomitant severe MR and TR, double surgical valve intervention is indicated in patients with acceptable risk (38). Current guidelines recommend combined TV surgery in patients with severe TR (Stages C and D) undergoing left-sided valve surgery and in patients with progressive TR (Stage B) with either (1) tricuspid annular dilatation (TAD, tricuspid annulus end-diastolic diameter >4.0 cm) or (2) prior signs and symptoms of right-sided HF (39). TR has been shown to progress after MV surgery over years in several studies and is associated with poor outcomes (8, 13, 40). Several studies have reported consistent results suggesting that ≥2+ TR should be treated concomitantly with MV surgery (8, 11, 40), with a hazard ratio of up to 2.5 for persistent heart failure (HF) after MV surgery if significant TR was present preoperatively (8).

While surgical data suggests that repairing the TV after addressing the predominant MV disease does not pose a significant additional surgical risk, some patients with mild preoperative TR might benefit from isolated MV surgery alone. This is evidenced by a study in 1,900 patients with degenerative MV disease with a structurally normal TV, of which 67 underwent a combined repair procedure. In those with mild preoperative TR, <20% of patients developed 2+ or greater TR at 3 years (13). In contrast, Kwak et al. showed that even with mild or moderate degrees of secondary TR, which is commonly not corrected at the time of left-sided valve surgery, may progress over time in ~25% of patients and result in reduced long-term functional outcome and survival (9).

Historically, surgical TV repair durability has been inconsistent, with recurrence rates of significant TR in up to 40% of cases depending on the technique performed (41). Ring annuloplasty has been shown to be superior to other repair techniques (DeVega suture annuloplasty, combined ring annuloplasty plus edge-to-edge suture or suture bicuspidization procedure), with >85% of patients being free from ≥2+ TR at 10 years (38). Importantly, reoperation for isolated TR after left-sided valve surgery is associated with a high perioperative mortality rates between 10 and 25% (42–44).

Several predictors for persistence or progression of TR have been reported and may assist with patient selection for a combined procedure. These include TAD (at end-diastole >40 mm diameter or 21 mm/m^2^ diameter on preoperative TTE; >70 mm diameter on direct intraoperative measurement of the inter-commissural distance), degree of RV dysfunction or remodeling, leaflet tethering height, pulmonary artery hypertension, AF, and intra-annular RV pacemaker or implantable cardioverter-defibrillator leads (10–12, 45–49). As it has been shown that addressing both significant MR and TR concomitantly leads to improvement in RV function (13), mirroring such results from the surgical literature with a combined TEER procedure is likely to improve patient outcomes.

### Clinical Outcomes of TEER for TR Based on Etiology of TR

Understanding the relative benefit of TEER for TR based on etiology is important in deciding when to intervene on the tricuspid valve with TEER. Recently, a study of 159 patients undergoing TTVr evaluated the impact of TR etiology on outcomes (50). Those with TR in the setting of severe MR made up almost 50% of the cohort, with the remaining patients TR etiology attributed to atrial fibrillation, pulmonary hypertension or chronic dialysis. TR secondary to MR or atrial fibrillation showed a lower primary endpoint of death, HF hospitalization or reintervention after intervention when compared to those with TR secondary to dialysis or pulmonary hypertension. Patients with dialysis-related TR had the greatest mortality with TTVr (33% at 1 year), while those with pulmonary hypertension had the highest rate of the primary endpoint of death, HF hospitalization, or reintervention. These results were consistent irrespective of whether patients underwent an isolated or combined TEER procedure (50). Thus, considering the underlying etiology of TR is important, and this study suggests that those with severe MR derive a significant benefit from TEER, and such patients should be considered for a combined TEER procedure.

## Evidence for Combined Mitral and Tricuspid Transcatheter Edge-to-Edge Repair

Although evidence is still limited, recently published registry data suggests that a combined TEER procedure is safe, effective and likely improves clinical outcomes (Table 1). In a small study of 27 high risk patients with severe MR and TR, undergoing a combined TEER procedure was associated with a lower rate of HF hospitalization, higher cardiac output, and reduction in N-terminal pro-B-type natriuretic peptide levels when compared to a matched control group undergoing MV TEER alone (51). In a subsequent larger study of 122 patients with severe MR and TR undergoing a combined TEER procedure from the TriValve Registry (52) demonstrating that not only did isolated tricuspid valve TEER was associated low procedural mortality and significant improvement in clinical outcomes, but that a combined TEER procedure was associated with a lower (16.4%) 1-year all-cause mortality when compared to matched patients from the TRAMI Registry (34.0%) (18) treated with isolated mitral TEER. On multivariate analysis, combined TEER was associated with a nearly 50% lower mortality rate (HR 0.52) after correcting for confounding variables (16). These promising data provide the basis for randomized trials to further evaluate the impact of combined TEER on clinical outcomes.

## Patient Selection for A Combined Teer Procedure: Tr Evolution After MV Teer

Recently, a large retrospective study of patients with baseline TR ranging from none/mild (41%), moderate (39%), and severe (19%) who underwent MitraClip for severe MR revealed several important findings pertaining to patient selection for a combined TEER procedure. First, TR improvement was associated with a lower rate of HF hospitalization at 2 years with a hazard ratio of 0.6. Second, patients with TAD (≥34 mm) at follow up had a higher rate of HF hospitalization. Third, TR was more likely to improve after MitraClip when the TAD decreased on follow up echocardiography. Fourth, that patients with atrial fibrillation were less likely to experience a decrease in TAD (and thus TR) and finally that MR > grade II at discharge was associated with lack of improvement in TR (17). Thus, when considering patients with severe MR and TR for a combined TEER procedure, those with TAD and atrial fibrillation should be favored for this approach. Interestingly, this study showed no association with baseline pulmonary hypertension and TR improvement, in contrast to two other smaller studies, one of which included patients with smaller TAD (53) and the other with larger TAD (54).

## Learning Curve for A Combined Teer Procedure

Becoming facile with TEER for treating MR is key to performing TEER for severe TR. Nevertheless, there remains a learning curve that needs to be overcome in order to optimize procedural success and improve patient outcomes. It has been shown that the learning curve when treating MR with MitraClip is steepest from 25 to 50 cases (55). In a retrospective review of 22 patients treated with combined TEER, procedure duration in the first tertile was significantly longer by 80 min (223 ± 13 vs. 143 ± 23 min) when compared to the third tertile. In addition, there was less residual TR comparing the beginning to the end of the study period (56). Although previous experience with MV TEER facilitates more rapid adoption of TV TEER by operators, imagers must also face a learning curve of tricuspid procedural guidance, which has its own unique challenges.

## Device Selection for Combined Teer

When planning TV TEER procedures, the choice of device can present a challenge given that these devices were developed for the mitral valve, whereas tricuspid valve leaflets are thinner and more fragile and severe TR tends to involve larger leaflet malcoaptation gaps. Some evidence from MV TEER has shown that the XTR clip leads to more SLDA and leaflet tearing (57), however the length of the XTR device is theoretically ideal when treating severe TR as most patients have malcoaptation gaps. The use of MitraClip XTR vs. NTR devices has been shown to achieve a higher procedural success rate (TR ≤ 2+ 80% vs. 70%), was able to treat TR with larger coaptation defects including those with torrential TR and lead to a greater reduction in TR. Importantly, the SLDA rates were similar (5%) (58) to those in the TRILUMINATE trial (4).

The MitraClip™ G4 system offers 2 new clip sizes (NTW and XTW) which measure 6 mm in the center, 50% wider than the 4-mm width of the NT/XT clip. The G4's larger grasping capability in addition to the option for independent leaflet grasping would be advantageous for treating TR, as recently reported (59, 60). Furthermore, the dedicated TriClip G4 system was recently given CE Mark and Health Canada approval for use in treating severe TR. Head-to-head studies of the TriClip and PASCAL systems for combined or isolated TV TEER awaits further investigation.

## Advantages of A Combined Teer Procedure

There are several advantages in considering a combined TEER procedure. First, a combined approach closely mirrors what is recommended in the guidelines for surgical intervention for TR in the setting of severe MR (39). Second, it avoids a second invasive procedure using general anesthesia in elderly and potentially frail patients. Third, it appears to reduce HF hospitalization and mortality, however a randomized clinical trial is clearly needed in this realm to better answer these and other important clinical questions. Finally, a combined procedure may reduce costs aside from avoiding a second procedure if the operator uses the same delivery system in treating both valves. Conversely, staged TV TEER procedures for only those whose TR persists after 1 month avoids unnecessary procedures. While some TEER procedures for combined TR and MR can be accomplished with the MitraClip guide (58), an advantage is offered by using the dedicated TriClip guide, as is being used in the TRILUMINATE Trial. Use of the MitraClip guide in performing TV TEER and conversely, the TriClip guide in performing MV TEER is off label. One perceived advantage of the PASCAL system is that it circumvents the need for different TEER guide catheters for treating both the MV and TV, however data for combined TEER with this device is sparse.

## Beyond Combined Teer

Although currently the most common interventional technique, TEER has anatomical limitations for the treatment of every patient with MR and TR. In future, a tailored approach using an expanded toolbox will allow more nuanced device selection based on valve anatomy and disease stage. Combined valve repair (TEER, annuloplasty, chordal implants) and replacement, or combined valve replacement may become a reality with advances in device development and procedural technique. Furthermore, future studies are needed to identify patients who will benefit the most from this treatment, the optimal anatomic features and standardized procedural success criteria associated with positive clinical outcomes.

## Discussion

As residual significant TR after MV TEER is an independent predictor for increased mortality, developing transcatheter therapies and having a more robust understanding of which patients will benefit from a combined TEER procedure is important. Small studies have demonstrated that a combined approach is safe, effective, and has shown promising short-term results, however randomized clinical trials are needed in this realm, especially regarding durability and persistent improvement in outcomes. Recent data has shed additional light on the deleterious effects of residual TR after MV TEER, including the implications of tricuspid annular dimensions and concomitant atrial fibrillation which will help guide patient selection for a combined procedure. There are several advantages to considering a combined procedure, both from clinical and cost perspectives. While there is a learning curve associated with both MV and TV TEER, the recent CE mark of the TriClip G4 and PASCAL systems will facilitate TV TEER in clinical practice. Ultimately, patients with concomitant severe MR and TR who are not ideal surgical candidates should be considered for a combined TEER procedure, using the available data to determine their suitability for this attractive therapeutic approach.

## Author Contributions

All authors listed have made a substantial, direct and intellectual contribution to the work, and approved it for publication.

## Conflict of Interest

NF has received speaker honoraria from Abbott Vascular and is a consultant for Edwards Lifesciences. The remaining authors declare that the research was conducted in the absence of any commercial or financial relationships that could be construed as a potential conflict of interest.

## Publisher's Note

All claims expressed in this article are solely those of the authors and do not necessarily represent those of their affiliated organizations, or those of the publisher, the editors and the reviewers. Any product that may be evaluated in this article, or claim that may be made by its manufacturer, is not guaranteed or endorsed by the publisher.
